# Genetic variants in the circadian rhythm pathway as indicators of prostate cancer progression

**DOI:** 10.1186/s12935-019-0811-4

**Published:** 2019-04-05

**Authors:** Chia-Cheng Yu, Lih-Chyang Chen, Chih-Yung Chiou, Yu-Jia Chang, Victor C. Lin, Chao-Yuan Huang, I-Ling Lin, Ta-Yuan Chang, Te-Ling Lu, Cheng-Hsueh Lee, Shu-Pin Huang, Bo-Ying Bao

**Affiliations:** 1 Division of Urology, Department of Surgery, Kaohsiung Veterans General Hospital, Kaohsiung, 813 Taiwan; 2 Department of Urology, School of Medicine, National Yang-Ming University, Taipei, 112 Taiwan; 3 Department of Pharmacy, Tajen University, Pingtung, 907 Taiwan; 4 Department of Medicine, Mackay Medical College, New Taipei City, 252 Taiwan; 5 Department of Gastroenterology and Hepatology, Chang Gung Memorial Hospital, Taoyuan, 333 Taiwan; 6 Graduate Institute of Clinical Medicine, College of Medicine, Taipei Medical University, Taipei, 110 Taiwan; 7 Department of Surgery, School of Medicine, College of Medicine, Taipei Medical University, Taipei, 110 Taiwan; 8 Division of General Surgery, Department of Surgery, Taipei Medical University Hospital, Taipei Medical University, Taipei, 110 Taiwan; 9 Cancer Research Center, Taipei Medical University Hospital, Taipei Medical University, Taipei, 110 Taiwan; 10 Department of Urology, E-Da Hospital, Kaohsiung, 824 Taiwan; 11 School of Medicine for International Students, I-Shou University, Kaohsiung, 840 Taiwan; 12 Department of Urology, National Taiwan University Hospital, College of Medicine, National Taiwan University, Taipei, 100 Taiwan; 13 Department of Urology, National Taiwan University Hospital Hsin-Chu Branch, Hsinchu, 300 Taiwan; 14 Department of Medical Laboratory Science and Biotechnology, College of Health Sciences, Kaohsiung Medical University, Kaohsiung, 807 Taiwan; 15 Department of Occupational Safety and Health, China Medical University, Taichung, 404 Taiwan; 16 Department of Pharmacy, China Medical University, Taichung, 404 Taiwan; 17 Department of Urology, Kaohsiung Medical University Hospital, Kaohsiung, 807 Taiwan; 18 Department of Urology, Faculty of Medicine, College of Medicine, Kaohsiung Medical University, Kaohsiung, 807 Taiwan; 19 Graduate Institute of Medicine, College of Medicine, Kaohsiung Medical University, Kaohsiung, 807 Taiwan; 20 Institute of Biomedical Sciences, National Sun Yat-sen University, Kaohsiung, 804 Taiwan; 21 Sex Hormone Research Center, China Medical University Hospital, Taichung, 404 Taiwan; 22 Department of Nursing, Asia University, Taichung, 413 Taiwan

**Keywords:** Circadian rhythm, Prostate cancer, Progression, Single nucleotide polymorphism, *NPAS2*

## Abstract

**Background:**

To determine the association between circadian pathway genetic variants and the risk of prostate cancer progression.

**Methods:**

We systematically evaluated 79 germline variants in nine circadian pathway genes in a cohort of 458 patients with localized prostate cancer as the discovery phase. We then replicated the significant findings in another cohort of 324 men with more advanced disease. The association of each variant with prostate cancer progression was evaluated by a log-rank test and Cox regression.

**Results:**

A single nucleotide polymorphism of the neuronal PAS domain protein 2 (*NPAS2*) gene (rs6542993 A>T) was found to be associated with a significantly higher risk of disease progression in both localized (*P* = 0.001) and advanced (*P* = 0.039) prostate cancer cases. In silico analysis revealed decreased expression levels of *NPAS2* in carriers of the T allele of rs6542993 compared with those carrying the A allele. Consistently, downregulation of *NPAS2* expression was associated with more aggressive prostate cancer and poor progression-free survival (log-rank *P* = 0.002).

**Conclusions:**

The *NPAS2* rs6542993 polymorphism may be a promising biomarker, and may shed light on the pathways that govern prostate cancer progression.

**Electronic supplementary material:**

The online version of this article (10.1186/s12935-019-0811-4) contains supplementary material, which is available to authorized users.

## Background

The circadian rhythm is driven by an internal biological clock, which allows organisms to sustain an approximate 24-h cycle of physiological activities such as the sleep–wake cycle, basal metabolism, hormone production, and immunity [1]. The core circadian clock is mainly generated through a series of transcriptional/translational feedback loops. In the morning, clock circadian regulator (CLOCK) and neuronal PAS domain protein 2 (NPAS2) form heterodimers with aryl hydrocarbon receptor nuclear translocator like (ARNTL), bind to E-box enhancer elements, and activate the transcription of period circadian regulators (PER1, PER2, PER3) and cryptochrome circadian regulators (CRY1, CRY2) [2–5]. Late in the day, PERs and CRYs heterodimerize and activate a negative feedback loop, which directly suppresses transcriptional activity of the CLOCK/ARNTL complex. The suppression of CLOCK/ARNTL is released through the degradation of PERs and CRYs by casein kinase 1 epsilon (CSNK1E) and ubiquitin-mediated pathways [6]. After PERs and CRYs are degraded, the entire cycle repeats with a periodicity of approximately 24 h.

Shift-work, sleep deprivation, jet lag, and light exposure at night potentially cause circadian disruption, which has been linked to the risk of various diseases such as diabetes, depression, cardiovascular problems, and cancer [7–10]. In particular, the International Agency for Research on Cancer classified shift-work with circadian disruption as a probable human carcinogen (Group 2A) [11]. A few candidate gene studies have examined the associations between circadian genes and several cancers [12–16], including prostate cancer. The association between circadian gene variants and prostate cancer risk has been described in several case–control study in Chinese and Caucasian population [13, 17]. However, an alternative study failed finding any association of 96 variants across 12 circadian-related genes with fatal prostate cancer using three patient cohorts [18]. Therefore, this gene-disease association remains inconsistent, and very few studies have assessed the prognostic values of these genes.

Based on accumulating evidence for a relationship between the circadian rhythm and cancer, we hypothesized that genetic variants in circadian pathway genes might have impacts on the prognosis of patients with prostate cancer. To test this hypothesis, we systematically evaluated the influence of 79 circadian gene variants on disease progression in patients with localized prostate cancers and then verified the findings in another group of patients with advanced prostate cancers.

## Methods

### Patient population and clinical data collection

A total of 458 patients with localized prostate cancer who underwent radical prostatectomy as initial treatment and 324 patients with advanced prostate cancer who were on androgen-deprivation therapy (ADT) were included in the present study. The recruitment process and patient characteristics were described previously [19–22]. In the localized prostate cancer cohort, biochemical recurrence (BCR) was defined as two consecutive prostate-specific antigen (PSA) level increments of > 0.2 ng/mL during an interval of > 3 weeks [23, 24]. In the advanced prostate cancer cohort, disease progression was defined as a serial rise, at least two rises over 1 week apart, in PSA over the nadir [25, 26]. Initiation of secondary hormone treatment for patients with a rising PSA level was also considered as a progression event. This study was performed in accordance with the approval procedures by the Institutional Review Board of Kaohsiung Medical University Hospital, and written informed consent was obtained from all patients.

### Single nucleotide polymorphism (SNP) selection and genotyping

TagSNPs were chosen from nine circadian-related genes (*ARNTL*, *CLOCK*, *CRY1*, *CRY2*, *CSNK1E*, *NPAS2*, *PER1*, *PER2*, and *PER3*) using the Tagger pairwise method [27] based on an *r*^2^ value of 0.8 or higher and a minor allele frequency of at least 0.2 in the HapMap Chinese Han population [28]. Ultimately, a total of 96 SNPs were selected for genotyping. Genomic DNA was extracted from peripheral blood samples using the QIAamp DNA Blood Mini Kit (Qiagen, Valencia, CA, USA). Genotyping was performed as described previously [29] using Agena Bioscience iPLEX matrix-assisted laser desorption/ionization time-of-flight mass-spectrometry technology at the National Center for Genome Medicine, Taiwan. Any SNP that failed at assay design (*N* = 7), deviated from Hardy–Weinberg equilibrium (*P* < 0.01, *N* = 5), or fell below a genotyping call rate of 0.85 (*N* = 3) was removed, leaving 79 SNPs for further analysis. The average genotype call rate was 99.2%, and the concordance rate was 100% among 10 duplicated samples.

### Bioinformatics analysis

We annotated the regulatory potential of the region adjoining the tagSNPs using HaploReg v4.1 [30] and ENCODE [31]. The GENe Expression VARiation (Genevar) database was used to identify potential SNP-gene expression quantitative trait loci (eQTL) associations within a locus [32]. The prognostic effect of *NPAS2* on prostate cancer was analysed using datasets from Memorial Sloan Kettering Cancer Center (MSKCC) Prostate Oncogenome Project [33].

### Statistical analysis

Kaplan–Meier analysis with the log-rank test was first used to assess the association of time to disease progression with each tagSNP under dominant, recessive, and additive models of inheritance. Since many tagSNPs were analysed, we conducted bootstrap resampling [34] to internally validate the significance of the tagSNPs by performing 1000 bootstrap runs. The risk of disease progression was estimated using the hazard ratios (HRs) and 95% confidence intervals (CIs) obtained by multivariate Cox regression adjusting for age, PSA level at diagnosis, pathologic Gleason score, and pathologic stage in the localized prostate cancer cohort [35], or adjusting for age, PSA level at ADT initiation, biopsy Gleason score, clinical stage, PSA nadir, and treatment modality in the advanced prostate cancer cohort [36]. Nonparametric analysis of variance followed by post hoc multiple comparison tests were applied to compare the level of *NPAS2* expression with clinical characteristics of the patients. All statistical analyses were performed using Statistical Package for the Social Sciences (SPSS) software version 19.0.0 (IBM, Armonk, NY, USA), and a two-sided *P* value of < 0.05 was considered statistically significant.

## Results

The clinical characteristics of the study groups are presented in Table 1. The median age of the patients in the localized prostate cancer cohort was 66 years. Over a median follow-up time of 54 months, 184 (40.2%) of the patients experienced a disease relapse. The median age of patients in the advanced prostate cancer cohort was 72 years, and 296 (91.4%) patients progressed to castration-resistant prostate cancer during the median follow-up of 93 months.

**Table 1:** Clinical characteristics of the study cohorts

Characteristic	
Localized prostate cancer cohort	
Patients, n	458
Age at diagnosis	
Median, years (IQR)	66 (61–70)
PSA at diagnosis	
Median, ng/mL (IQR)	11.1 (7.1–17.5)
Pathologic Gleason score, n (%)	
< 7	160 (35.3)
7	232 (51.2)
> 7	61 (13.5)
Pathologic stage, n (%)	
T1/T2	303 (67.2)
T3/T4/N1	148 (32.8)
M1	0 (0.0)
Disease progression	
No	274 (59.8)
Yes	184 (40.2)
Advanced prostate cancer cohort	
Patients, n	324
Age at diagnosis	
Median, years (IQR)	72 (66–78)
PSA at ADT initiation	
Median, ng/mL (IQR)	32.6 (9.4–123.4)
Biopsy Gleason score at diagnosis, n (%)	
< 7	70 (22.2)
7	131 (41.5)
> 7	115 (36.4)
Clinical stage at diagnosis, n (%)	
T1/T2	92 (28.6)
T3/T4/N1	103 (32.0)
M1	127 (39.4)
PSA nadir	
Median, ng/mL (IQR)	0.12 (0.01–1.08)
Treatment modality	
ADT as primary treatment	138 (42.7)
ADT for post RP PSA failure	39 (12.1)
ADT for post RT PSA failure	6 (1.9)
Neoadjuvant/adjuvant ADT with RT	101 (31.3)
Others	39 (12.1)
Disease progression	
No	28 (8.6)
Yes	296 (91.4)

IQR, interquartile range; PSA, prostate-specific antigen; ADT, androgen-deprivation therapy; RP, radical prostatectomy; RT, radiation therapy

**Fig. 1:**
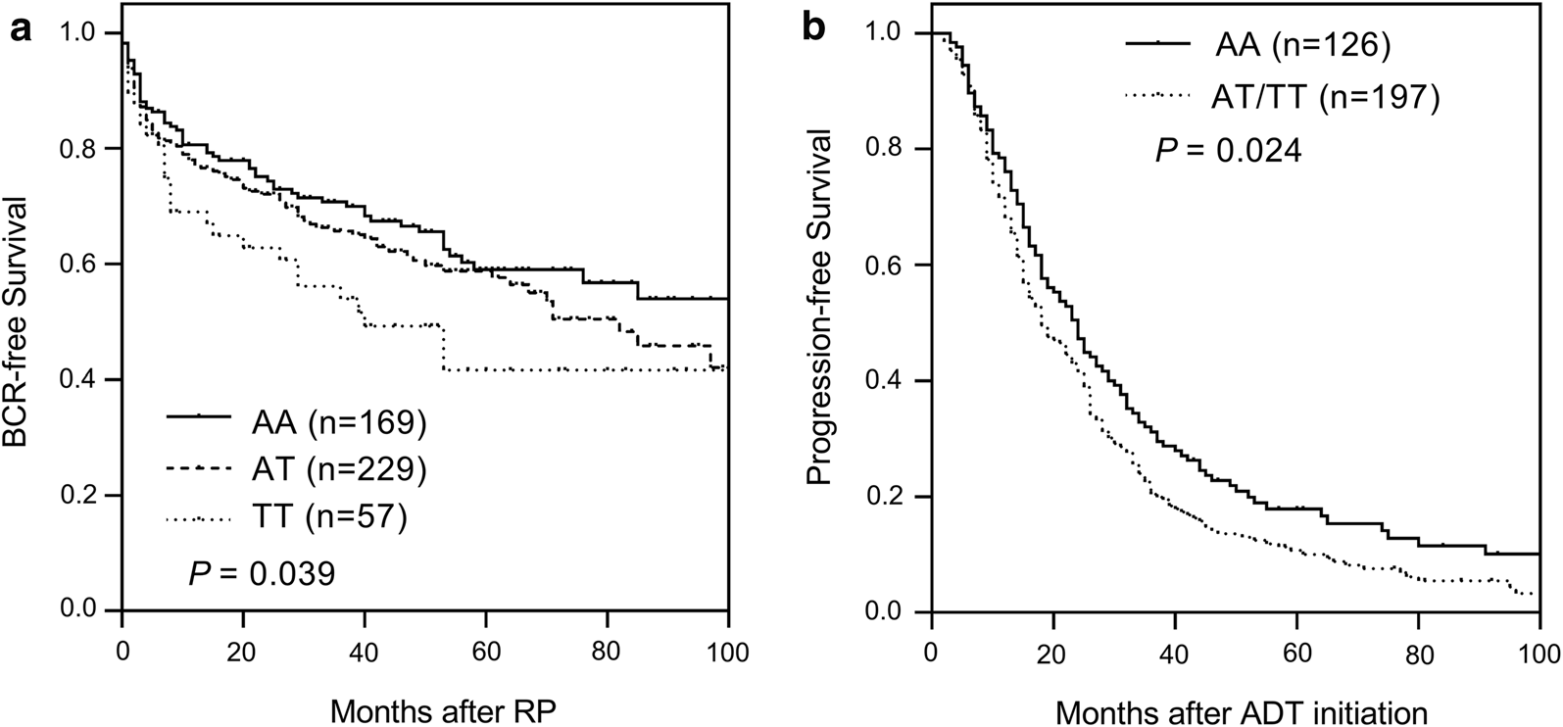
Impact of the SNP *NPAS2* rs6542993 on prostate cancer progression. Kaplan–Meier estimates of **a** biochemical recurrence (BCR)-free survival in localized prostate cancer patients who received radical prostatectomy, and **b** progression-free survival in patients with advanced prostate cancer who received androgen-deprivation therapy (ADT) according to *NPAS2* rs6542993 genotypes

In the localized prostate cancer cohort, we screened 79 SNPs within nine circadian-related genes. *NPAS2* rs6542993 was significantly associated with BCR (*P* = 0.039, Table 2 and Fig. 1a), while no significant association was detected for the other selected variants (Additional file 1: Table S1). The prognostic value of rs6542993 remained significant with 1000 bootstrap resampling (*P* = 0.042, Table 2), and in multivariate analysis after adjusting for age, PSA level at diagnosis, pathologic Gleason score, and stage (HR 1.50, 95% CI 1.18–1.91, *P* = 0.001; Table 2), providing support for the validity of this result. Interestingly, *NPAS2* rs6542993 was also found to be associated with an increased risk of progressive disease (HR 1.32, 95% CI 1.01–1.71, *P* = 0.032; Table 3 and Fig. 1b) after adjusting for known clinicopathological variables that are associated with advanced prostate cancer, confirming *NPAS2* rs6542993 as a biomarker for prostate cancer progression.

**Table 2:** Association of *NPAS2* rs6542993 with BCR in localized prostate cancer patients treated with RP

Gene	SNP	Genotype	No. of patients	No. of events	5-year survival rate (%)	*P* ^a^	*P* ^b^	HR (95% CI)	*P* ^c^
*NPAS2*	rs6542993	AA	169	63	59.0			1.00	
		AT	229	90	58.8			1.40 (0.99–1.98)	0.059
		TT	57	29	41.6			2.34 (1.45–3.79)	*0.001*
		AT/TT vs AA				0.164	0.153	1.53 (1.10–2.14)	*0.012*
		TT vs AA/AT				*0.034*	*0.028*	1.92 (1.25–2.94)	*0.003*
		Trend				*0.039*	*0.042*	1.50 (1.18–1.91)	*0.001*

PSA, prostate-specific antigen; RP, radical prostatectomy; SNP, single nucleotide polymorphism; HR, hazard ratio; 95% CI, 95% confidence interval

*P* < 0.05 are in italicsface

^a^*P* values were calculated using the log-rank test

^b^*P* values were calculated after correcting for multiple tests by 1000 bootstrap resampling

^c^HRs were adjusted for age, PSA at diagnosis, pathologic Gleason score, and pathologic stage

**Table 3:** Association of *NPAS2* rs6542993 with disease progression in advanced prostate cancer patients treated with ADT

Gene	SNP	Genotype	No. of patients	No. of events	5-year survival rate (%)	*P* ^a^	HR (95% CI)	*P* ^b^
*NPAS2*	rs6542993	AA	126	109	17.9		1.00	
		AT	150	143	10.4		1.44 (1.09–1.90)	*0.010*
		TT	47	43	10.9		1.05 (0.73–1.53)	0.782
		AT/TT vs AA				*0.024*	1.32 (1.01–1.71)	*0.039*
		TT vs AA/AT				0.362	0.87 (0.62–1.22)	0.419
		Trend				0.284	1.09 (0.92–1.28)	0.339

ADT, androgen-deprivation therapy; SNP, single nucleotide polymorphism; HR, hazard ratio; 95% CI, 95% confidence interval; PSA, prostate-specific antigen

*P* < 0.05 are in italicsface

^a^*P* values were calculated using the log-rank test

^b^HRs were adjusted for age, PSA at ADT initiation, biopsy Gleason score, clinical stage, PSA nadir, and treatment modality

**Fig. 2:**
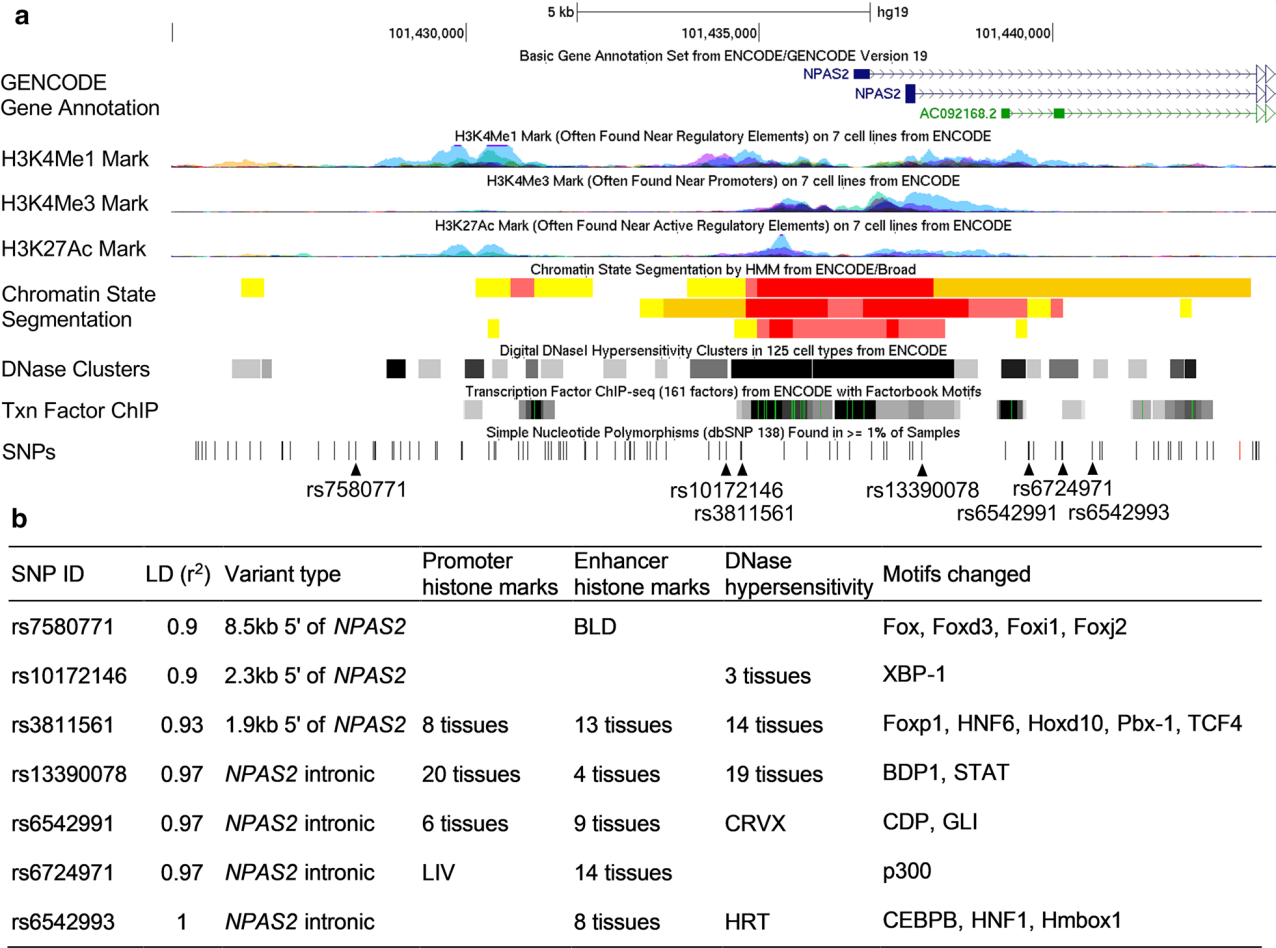
**a** ENCODE data for the linkage disequilibrium (LD) block containing *NPAS2* rs6542993. H3K4Me1, H3K4Me3, and H3K27Ac tracks show the levels of enrichment of the mono-methylation of lysine 4, tri-methylation of lysine 4, and acetylation of lysine 27 of the H3 histone protein, respectively, across the genome as determined by chromatin immunoprecipitation sequencing (ChIP-seq) assays. These marks are thought to be associated with enhancer and promoter regions. Chromatin State Segmentation track displays chromatin state segmentations by integrating the ChIP-seq data using a Hidden Markov Model for HepG2 hepatocellular carcinoma cells, HMEC normal mammary epithelial cells, and NHLF normal lung fibroblast cells. The chromatin state regions predicted for promoters and enhancers are highlighted. The DNase clusters track shows DNase hypersensitivity areas. The Tnx factor track shows regions of transcription factor binding to DNA as assayed by ChIP-seq experiments. **b** Regulatory annotation of variants within the LD block containing *NPAS2* rs6542993. In the LD block with the tagSNP rs6542993, there are strong enrichments of promoter and enhancer marks among the several different cell types tested. In addition, multiple regulatory motifs are predicted to be affected by the linked variants

**Fig. 3:**
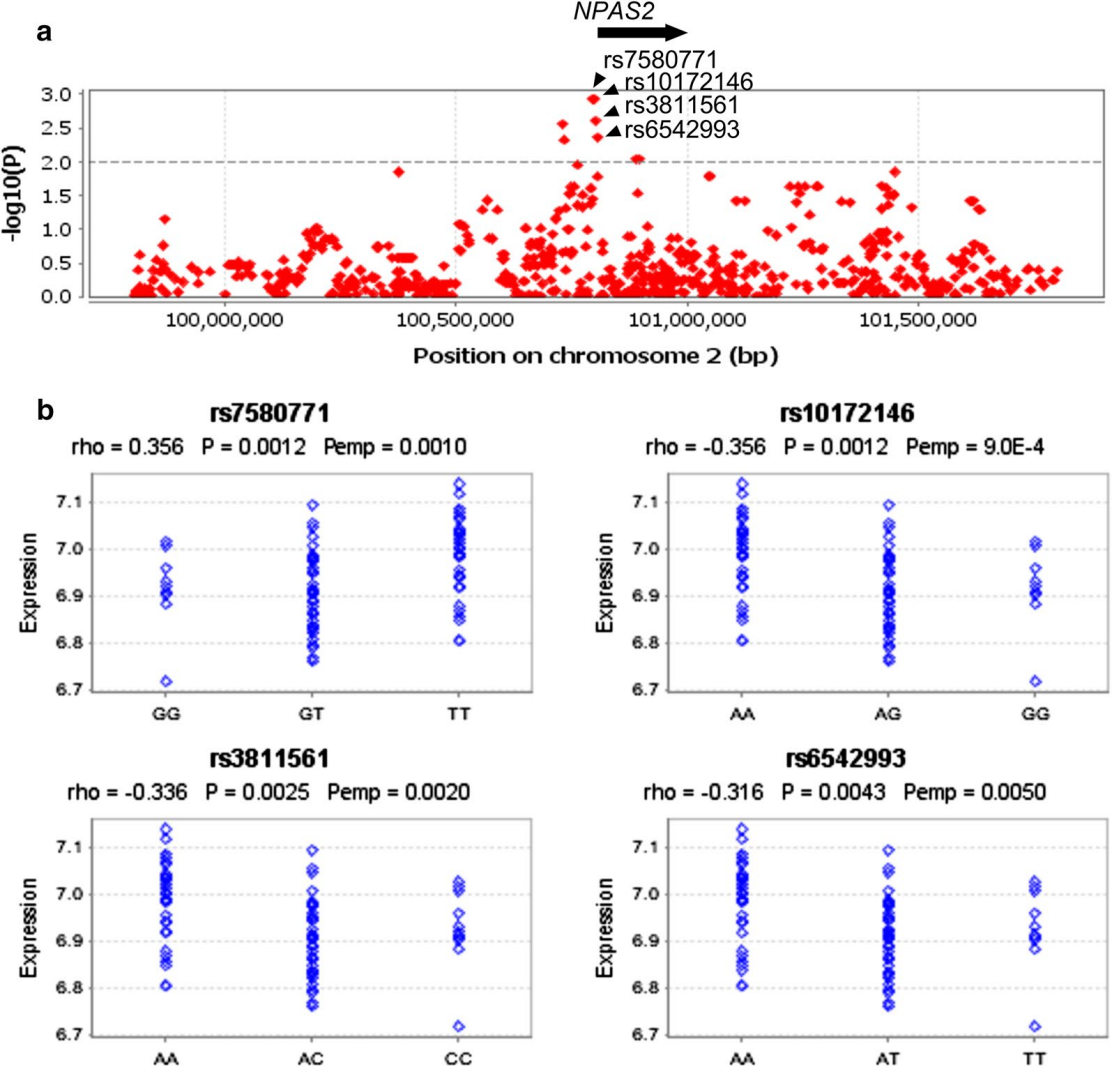
Expression quantitative trait loci (eQTL) analyses targeting the *NPAS2* locus with data of the HapMap CHB population. **a** eQTL SNPs are visualized as a regional plot (2 Mb), where a dotted line represents the *P* value threshold (0.01). **b** Best candidate eQTL SNPs after running 10,000 permutations. rho, Spearman’s rank correlation coefficient; P, *P* value; Pemp, adjusted *P* value after running 10,000 permutations. Note that *NPAS2* is located between coordinates 100,803,039 and 100,979,719 according to the NCBI36/Ensembl 50 assembly

We annotated all correlated variants within the linkage disequilibrium (LD) block (*r*^2^ ≥ 0.9) of rs6542993 with respect to regulatory elements, and predicted functional motifs catalogued in ENCODE and HaploReg (Fig. 2). All SNPs coincided with promoter or enhancer elements: five SNPs are located in DNase hypersensitivity domains and are predicted to alter transcription factor-binding motifs, suggesting that these SNPs could influence gene expression. Genevar analysis using HapMap data to investigate the association of these SNPs with *NPAS2* expression demonstrated that rs6542993 and linked SNPs coincide with a probable eQTL and are the best candidates to influence *NPAS2* expression. The risk allele T of rs6542993 was associated with a decreased *NPAS2* expression level in the HapMap Han Chinese in Beijing (CHB) population (empirical *P* = 0.005, Fig. 3).

**Fig. 4:**
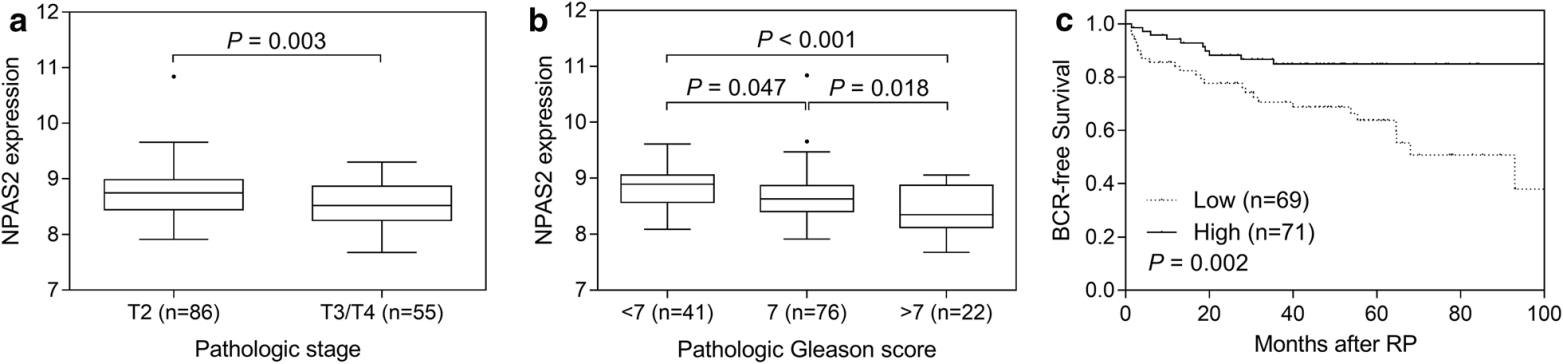
Negative correlation of *NPAS2* mRNA expression with prostate cancer progression. The associations between *NPAS2* expression and prostate cancer aggressiveness were analysed using data of the MSKCC Prostate Oncogenome Project. More advanced prostate cancers, pathologic stage T3/T4 vs. T2 (**a**), and pathologic Gleason score ≥ 7 vs. < 7 (**b**) were associated with significantly lower mRNA expression levels of *NPAS2*. **c** Kaplan–Meier curves of biochemical recurrence (BCR)-free survival according to the expression of *NPAS2*. Patients were dichotomized based on the median expression level of *NPAS2*

We further evaluated the association of *NPAS2* expression with prostate cancer outcome using the MSKCC Prostate Oncogenome Project data. Consistently, the *NPAS2* gene expression level was significantly lower in cases of more aggressive forms of prostate cancer (*P* ≤ 0.003, Fig. 4a, b), and a low expression level of *NPAS2* was associated with poor BCR-free survival in patients with prostate cancer (*P* = 0.002, Fig. 4c).

## Discussion

We conducted a two-stage study to investigate the effects of common germline genetic variants in the circadian pathway on the prognosis of prostate cancer patients. Among the 79 variants assessed in nine genes, *NPAS2* rs6542993 was identified as an independent prognostic factor for prostate cancer progression across both cohorts. In addition, bioinformatics analysis provided further evidence that rs6542993 is an eQTL that affects the expression of *NPAS2*, and down-regulation of *NPAS2* expression was correlated with a shorter progression-free survival of prostate cancer patients. Overall, these findings highlight the importance of *NPAS2* in prostate cancer progression.

The risk variant rs6542993 is located in the intronic region of *NPAS2*, displaying histone marks with transcriptional enhancer activity. In addition to rs6542993, multiple SNPs in high LD were also identified as significant eQTLs for *NPAS2*. In fact, rs10172146, a SNP in high LD (*r*^2^ = 0.9) with the risk allele, showed a strong association with *NPAS2* expression. The risk allele rs6542993 T was associated with reduced levels of *NPAS2* expression. Only a few studies have examined the role of circadian gene variants on prostate cancer risk, with some suggestive general associations reported [13, 17, 18]. *NPAS2* has consistently emerged as one of the top genes with the greatest contribution to the observed statistical association between the circadian pathway and risk of prostate cancer in two meta-analyses of data from genome-wide association studies [37, 38]. *NPAS2* is suggested to be a putative tumour suppressor playing an important role in transcriptional suppression of the protooncogene c-Myc [39], DNA damage response [40], and cell cycle control by regulating diverse downstream genes [41]. In addition, *NPAS2* has been identified as a prognostic biomarker in breast and colorectal cancers [42, 43], in which reduced *NPAS2* expression was associated with decreased disease-free and overall survival rates in patients with breast cancer [43], in line with our present results in patients with prostate cancer. Silencing *NPAS2* expression promoted the proliferation, invasion, and wound healing abilities of colorectal cancer cells [42], indicating a crucial role of *NPAS2* in tumour growth and metastasis. We found no association between *NPAS2* rs6542993 polymorphism and prostate cancer risk when comparing our patient population to 1500 healthy controls from the Taiwan Biobank (data not shown). The *NPAS2* gene expression levels also did not vary significantly when comparing prostate cancers and normal tissues using the MSKCC Prostate Oncogenome Project data. These data suggested that *NPAS2* might play a role in prostate cancer progression but not in prostate cancer initiation. However, the functional roles of *NPAS2* in prostate cancer progression remain largely unclear and need further investigation.

The modest sample size of both cohorts included in this study did not allow for optimal statistical power to detect associations; therefore, the observed *P* values could not reach a more stringent significance level to avoid multiple comparisons. However, the association between *NPAS2* rs6542993 and prostate cancer progression was replicated across two independent and different types of cohorts, which largely reduces the chance of false-positive findings. In addition, functional analyses support the role of *NPAS2* rs6542993 in patient prognosis. Since the tagSNPs evaluated in this study were selected based on haplotype diversities, the linked causal variants remain to be determined. Since circadian genes are expressed in a circadian manner, the timing of specimen collection and the measurements of genetic variants and gene expression should be consistent in the same population. Another potential limitation of the study is that our findings may not be generalized to other ethnic groups since the majority of patients in both cohorts were Taiwanese. However, this may also be a benefit of our study in reducing the effects of population heterogeneity to more clearly detect an association. Nevertheless, further independent, larger, and inter-ethnic studies, as well as functional experiments are needed to validate our findings.

## Conclusions

This is the first attempt to systematically evaluate the effect of genetic variants in the circadian pathway on prostate cancer progression. *NPAS2* rs6542993 was consistently associated with disease progression after adjusting for clinical confounders in two independent prostate cancer cohorts. This SNP might affect prostate cancer progression by reducing the expression level of *NPAS2*. Thus, our findings might help to improve understanding of the interaction of circadian dysfunction and prostate cancer progression, and can provide a promising biomarker toward realizing the personalized management of patients with prostate cancer.

## Additional file

**Additional file 1: Table S1.** Genotyped SNPs and the *P* values of their association with biochemical recurrence after radical prostatectomy.
