# Supplementary material for: Genetic variants in the circadian rhythm pathway as indicators of prostate cancer progression
Source: Cancer Cell Int. 2019 Apr 5;19:87. doi: 10.1186/s12935-019-0811-4 (PMC6451277; doi:10.1186/s12935-019-0811-4)
Supplement: Supplementary file 1 — Additional file 1: Table S1. Genotyped SNPs and the P values of their association with biochemical recurrence after radical prostatectomy. [file 12935_2019_811_MOESM1_ESM.doc]

**Table S1 Genotyped SNPs and the *P* values of their association with biochemical recurrence after radical prostatectomy**

| Gene | SNP ID | Chromosome | Position | BCR | | |
| --- | --- | --- | --- | --- | --- | --- |
|  |  |  |  | Additive | Dominant | Recessive |
| *PER3* | rs10864315 | chr1 | 7772668 | 0.555 | 0.976 | - |
| *PER3* | rs17031578 | chr1 | 7781778 | 0.833 | 0.804 | 0.367 |
| *PER3* | rs707467 | chr1 | 7784271 | 0.207 | 0.340 | 0.264 |
| *PER3* | rs10746473 | chr1 | 7800643 | 0.554 | 0.452 | 0.853 |
| *NPAS2* | rs6542993 | chr2 | 100807096 | **0.039** | 0.164 | **0.034** |
| *NPAS2* | rs3849377 | chr2 | 100814358 | 0.892 | 0.950 | 0.657 |
| *NPAS2* | rs3860456 | chr2 | 100816744 | 0.579 | 0.184 | 0.370 |
| *NPAS2* | rs6542994 | chr2 | 100817953 | 0.593 | 0.737 | 0.567 |
| *NPAS2* | rs10193688 | chr2 | 100824423 | 0.769 | 0.785 | 0.851 |
| *NPAS2* | rs6722909 | chr2 | 100827527 | 0.662 | 0.570 | - |
| *NPAS2* | rs7582455 | chr2 | 100838139 | 0.806 | 0.644 | - |
| *NPAS2* | rs7598826 | chr2 | 100839866 | 0.432 | 0.579 | 0.453 |
| *NPAS2* | rs930309 | chr2 | 100850966 | 0.175 | 0.342 | 0.190 |
| *NPAS2* | rs6542996 | chr2 | 100852259 | 0.857 | 0.950 | 0.545 |
| *NPAS2* | rs41349646 | chr2 | 100852564 | 0.955 | 0.906 | 0.781 |
| *NPAS2* | rs12472319 | chr2 | 100853889 | 0.970 | 0.673 | 0.613 |
| *NPAS2* | rs2871389 | chr2 | 100861606 | 0.402 | 0.477 | 0.495 |
| *NPAS2* | rs17024926 | chr2 | 100872434 | 0.488 | 0.263 | 0.885 |
| *NPAS2* | rs12712083 | chr2 | 100875331 | 0.646 | 0.764 | 0.158 |
| *NPAS2* | rs1369481 | chr2 | 100878391 | 0.780 | 0.634 | 0.800 |
| *NPAS2* | rs11123853 | chr2 | 100890908 | 0.982 | 0.089 | 0.052 |
| *NPAS2* | rs356655 | chr2 | 100895847 | 0.481 | 0.138 | 0.579 |
| *NPAS2* | rs7602455 | chr2 | 100903823 | 0.948 | 0.342 | 0.125 |
| *NPAS2* | rs3754675 | chr2 | 100916275 | 0.580 | 0.158 | 0.392 |
| *NPAS2* | rs3739005 | chr2 | 100932502 | 0.477 | 0.437 | 0.708 |
| *NPAS2* | rs3820787 | chr2 | 100932666 | 0.952 | 0.834 | 0.708 |
| *NPAS2* | rs4851386 | chr2 | 100933370 | 0.515 | 0.594 | 0.596 |
| *NPAS2* | rs876060 | chr2 | 100943396 | 0.263 | 0.570 | 0.102 |
| *NPAS2* | rs12613953 | chr2 | 100943810 | 0.448 | 0.463 | 0.608 |
| *NPAS2* | rs6747755 | chr2 | 100944890 | 0.555 | 0.949 | 0.133 |
| *NPAS2* | rs12622050 | chr2 | 100945886 | 0.248 | 0.602 | 0.094 |
| *NPAS2* | rs17025078 | chr2 | 100946008 | 0.484 | 0.276 | 0.741 |
| *NPAS2* | rs17025086 | chr2 | 100947940 | 0.996 | 0.602 | 0.598 |
| *NPAS2* | rs7340468 | chr2 | 100950018 | 0.953 | 0.574 | 0.499 |
| *NPAS2* | rs2305160 | chr2 | 100957736 | 0.161 | 0.215 | - |
| *NPAS2* | rs2305159 | chr2 | 100957875 | 0.723 | 0.552 | - |
| *NPAS2* | rs1542179 | chr2 | 100961667 | 0.750 | 0.733 | 0.129 |
| *NPAS2* | rs1542178 | chr2 | 100961907 | 0.703 | 0.503 | - |
| *NPAS2* | rs2278728 | chr2 | 100964744 | 0.797 | 0.857 | 0.305 |
| *NPAS2* | rs2278727 | chr2 | 100965109 | 0.429 | 0.648 | 0.352 |
| *NPAS2* | rs6719437 | chr2 | 100965503 | 0.939 | 0.986 | 0.821 |
| *NPAS2* | rs6719533 | chr2 | 100965519 | 0.098 | 0.097 | 0.288 |
| *NPAS2* | rs3754680 | chr2 | 100965950 | 0.784 | 0.460 | 0.539 |
| *NPAS2* | rs1867861 | chr2 | 100974473 | 0.242 | 0.529 | 0.150 |
| *NPAS2* | rs2305158 | chr2 | 100978483 | 0.734 | 0.658 | - |
| *NPAS2* | rs3739008 | chr2 | 100979000 | 0.278 | 0.210 | 0.850 |
| *PER2* | rs934945 | chr2 | 238819792 | 0.161 | 0.457 | 0.053 |
| *PER2* | rs11894535 | chr2 | 238841812 | 0.150 | 0.331 | 0.089 |
| *PER2* | rs2304676 | chr2 | 238844653 | 0.083 | 0.140 | 0.166 |
| *PER2* | rs6754875 | chr2 | 238857958 | 0.368 | 0.504 | 0.378 |
| *CLOCK* | rs2412648 | chr4 | 56015824 | 0.615 | 0.462 | 0.983 |
| *CLOCK* | rs10002541 | chr4 | 56089768 | 0.532 | 0.808 | 0.351 |
| *ARNTL* | rs7950226 | chr11 | 13274715 | 0.707 | 0.862 | 0.641 |
| *ARNTL* | rs11605776 | chr11 | 13275100 | 0.705 | 0.837 | 0.405 |
| *ARNTL* | rs10766074 | chr11 | 13275142 | 0.101 | 0.135 | 0.266 |
| *ARNTL* | rs4757142 | chr11 | 13282271 | 0.432 | 0.468 | 0.595 |
| *ARNTL* | rs10766076 | chr11 | 13297286 | 0.783 | 0.837 | 0.475 |
| *ARNTL* | rs7126796 | chr11 | 13297443 | 0.588 | 0.863 | - |
| *ARNTL* | rs12805304 | chr11 | 13301221 | 0.454 | 0.702 | 0.358 |
| *ARNTL* | rs10766077 | chr11 | 13306357 | 0.471 | 0.348 | 0.919 |
| *ARNTL* | rs11022769 | chr11 | 13308981 | 0.264 | 0.373 | 0.356 |
| *ARNTL* | rs16912743 | chr11 | 13310940 | 0.292 | 0.330 | - |
| *ARNTL* | rs1026071 | chr11 | 13321288 | 0.941 | 0.823 | 0.720 |
| *ARNTL* | rs2290036 | chr11 | 13336364 | 0.608 | 0.593 | - |
| *ARNTL* | rs1868049 | chr11 | 13340258 | 0.765 | 0.912 | 0.687 |
| *ARNTL* | rs3816358 | chr11 | 13348048 | 0.887 | 0.987 | - |
| *ARNTL* | rs4757151 | chr11 | 13348789 | 0.942 | 0.692 | 0.466 |
| *ARNTL* | rs11600996 | chr11 | 13352742 | 0.592 | 0.775 | 0.429 |
| *ARNTL* | rs12363415 | chr11 | 13355184 | 0.898 | 0.875 | - |
| *ARNTL* | rs10832030 | chr11 | 13356367 | 0.872 | 0.957 | 0.727 |
| *CRY2* | rs2292912 | chr11 | 45834264 | 0.953 | 0.779 | 0.805 |
| *CRY2* | rs1401419 | chr11 | 45836315 | 0.731 | 0.583 | 0.834 |
| *CRY2* | rs6798 | chr11 | 45861053 | 0.909 | 0.974 | 0.817 |
| *CRY1* | rs7134492 | chr12 | 105931935 | 0.508 | 0.419 | 0.985 |
| *CRY1* | rs11113181 | chr12 | 105992391 | 0.457 | 0.264 | 0.756 |
| *PER1* | rs3027188 | chr17 | 7989710 | 0.343 | 0.409 | 0.483 |
| *PER1* | rs3027178 | chr17 | 7993810 | 0.082 | 0.205 | 0.077 |
| *CSNK1E* | rs2075984 | chr22 | 37020835 | 0.698 | 0.653 | 0.875 |
| *CSNK1E* | rs135757 | chr22 | 37033599 | 0.369 | 0.203 | - |

*P* values for log-rank test.

*P* < 0.05 is in boldface.
